# Sleep Disturbances and Suicidality–A Longitudinal Analysis From a Representative Community Study Over 30 Years

**DOI:** 10.3389/fpsyt.2018.00320

**Published:** 2018-07-16

**Authors:** Wulf Rössler, Jules Angst, Vladeta Ajdacic-Gross, Helene Haker, Sofian Berrouiguet, Mariam Ujeyl, Nicholas Glozier, Michael P. Hengartner

**Affiliations:** ^1^Department of Psychiatry, Psychotherapy, and Psychosomatics, Universität Zurich, Zurich, Switzerland; ^2^Department of Psychiatry and Psychotherapy, Charité Universitätsmedizin, Berlin, Germany; ^3^Laboratory of Neuroscience, Institute of Psychiatry, Universidade de São Paulo, São Paulo, Brazil; ^4^5IMT Atlantique, Lab-STICC, UBL, EA 7479 SPURBO, Université de Bretagne Occidentale, Brest, France; ^5^Brain and Mind Centre, Sydney Medical School, University of Sydney, Sydney, NSW, Australia; ^6^Department of Applied Psychology, Zurich University of Applied Sciences, Winterthur, Switzerland

**Keywords:** epidemiology, sleep, suicidality, depression, insomnia

## Abstract

**Study objectives:** Associations between sleep problems and suicidality are increasingly acknowledged, but prospective data from well-controlled long-term community studies are lacking.

**Methods:** We analyzed data from a longitudinal cohort study with *n* = 591 young adults from Zurich, Switzerland, prospectively followed from 1979 (age 20/21 years) to 2008 (age 49/50 years). Twelve-month prevalence of various mental disorders, socio-environmental confounders and sleep problems were carefully assessed with semi-structured interviews at 7 assessment waves spanning overall a 30-year observation period. Interviews were conducted with the “Structured Psychopathological Interview and Rating of the Social Consequences of Psychological Disturbances for Epidemiology” (SPIKE). The 12-month prevalence of sleep problems was graded according to frequency and associated distress of reported symptoms. 12-month prevalence of suicidality was classified as either mild (transient suicidal ideation) or severe (self-harm, suicide attempts).

**Results:** Concurrently, and fully adjusted for several covariates, including mental disorders, relative to no sleep problems there was an odds ratio (OR) of *OR* = 1.9 (95% confidence interval 1.4–2.5), *OR* = 3.3 (2.5–4.4), and *OR* = 1.9 (1.3–2.8) for mild, moderate and severe sleep problems in association with suicidality. There was no evidence for a prospective effect of broad sleep problems on subsequent suicidality. Mild suicidality, but not severe suicidality, prospectively predicted subsequent broad sleep problems in the fully adjusted multivariate model (adjusted *OR* = 1.5; 1.1–1.9). Disturbed sleep initiation, a proxy for insomnia, significantly predicted subsequent suicidality (*OR* = 1.5; 1.1–1.9), whereas mild suicidality, but not severe suicidality, significantly predicted subsequent insomnia (*OR* = 1.5; 1.1–2.0).

**Conclusions:** Sleep problems and suicidality are longitudinally inter-related, which has important implications for clinical practice. Most importantly, the causal pathways appear to be bi-directional and independent of socio-demographics and concomitant mental disorders. More research is needed to examine the possible biopsychosocial etiological mechanisms linking suicidality to sleep problems.

## Introduction

Suicide is worldwide a major problem, though the rates vary considerably between countries (1, 2). Concerning burden of disease, the impact of suicide is comparable to the impact of cardio-vascular disease (2). Suicide attempts are 10 times more frequent than suicide and the lifetime prevalence rates for suicidal ideation lie around 40–50% in the general population (3). Since Emile Durkheim suicidality is regarded a social-cultural problem (4). Epidemiological surveys indeed indicate that apart from socio-demographic indicators as age and gender, low social support, childhood traumata and associated mental disorders play a major role in the onset and course of suicidality (5–7). Another risk factor associated with suicidality, which recently has been described, are sleep disturbances (8, 9). There is a growing body of evidence from clinical samples as well as community samples that such disturbances are in fact associated with an elevated risk for suicidality [for reviews, see references (10–12)]. Therefore, sleep is an important topic in the public mental health arena with a focus on preventive measures. It remains unclear, however, if sleep disturbances stand alone as a risk factor for suicidality or if suicidality has to be analyzed in a broader context of mental disorders. Suicidality occurs in the presence of mental disorders and both sleep disturbances and suicidal ideation are important features of diagnosed depression, i.e., there is a substantial overlap between suicidality and depression. However, previous research was either cross-sectional [e.g., (8, 13)] or did only focus on the prospective effect of sleep problems on subsequent suicidality, but not on the reverse association [e.g., (9, 14)]. Moreover, previous research has predominantly focused on insomnia, but not on sleep problems more generally [see for instance the review by Winsper and Tang (15)].

Theoretical explanations concerning the connection between sleep and suicidality are given less with respect to socio-cultural and/or psychological factors but rather with biological factors. As such, studies were conducted about sleep EEG irregularities or sleep disordered breathing to analyse this relationship (10). Other possible theoretical links between sleep disturbances and suicidality are sought for in the serotonin metabolism, as—aside from its possible role in mood disorders—serotonin has an important role in regulating the induction and maintenance of sleep (16). Other potential mechanisms focus on various dysfunctions of the HPA axis (17).

In a recent analysis from a large and representative epidemiological sample, it was demonstrated that sleep disturbances are highly prevalent, i.e., up to 25% among the young and middle-aged adults, and as such indeed represent a serious public mental health problem (18). Here we want to analyze the connection between sleep disturbances and suicidality in another community sample in which the participants have been assessed longitudinally over a 30-year time span. Previous analyses from this community sample were conducted to describe prevalence, course and comorbidity of insomnia with depression (19), excessive daytime sleepiness (20), and short sleep duration and obesity (21). In this paper, we test the following three hypotheses: (i) are broad sleep problems and suicidality bi-directionally related when the effect of common mental disorders is accounted for, (ii) do sleep problems indeed precede suicidality, and (iii) is there also evidence for a reverse association, that is, does suicidality precede sleep problems.

## Methods

### Participants and sampling procedure

**Figure 1:**
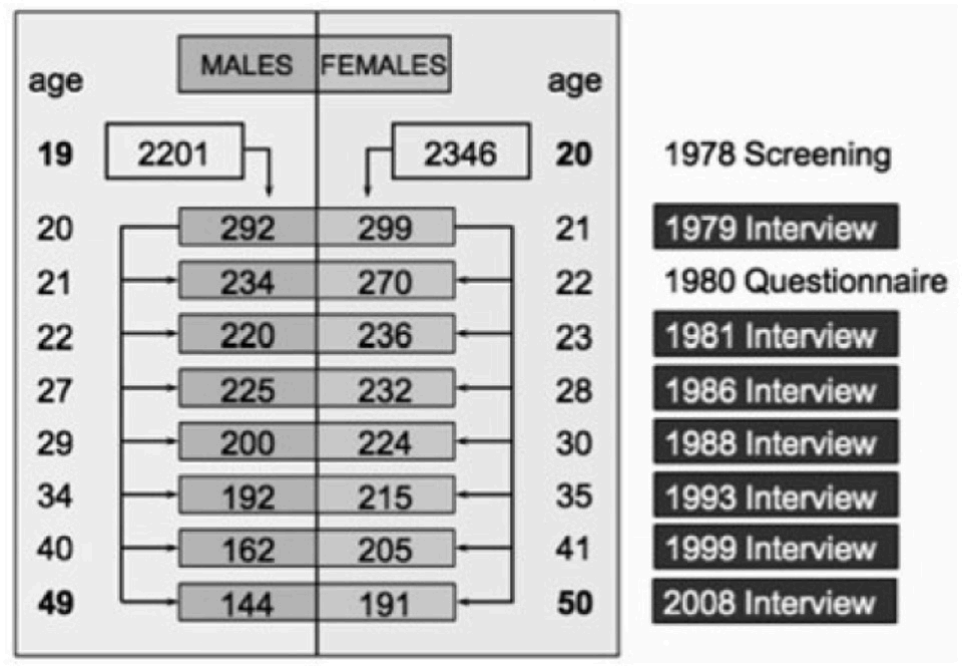
Participant flow.

The longitudinal Zurich Cohort Study comprised a cohort of 4,547 persons (2,201 males; 2,346 females) representative of the canton of Zurich in Switzerland, who were screened in 1978 with the Symptom Checklist 90-Revised (SCL-90-R) (22) when men were 19 and women 20 years old. In Switzerland, every male person must undertake a military screening test at the age of 19. Therefore, conscripts within a defined catchment area comprise its respective, complete male age group. With the consent of military authorities, but independent of their screening procedure, we randomly screened 50% of all conscripts of the canton of Zurich (refusal rate: 0.3%). Female participants were identified from the complete electoral register of the canton of Zurich. Again, 50% of them were randomly selected and received questionnaires by mail (refusal rate: 25%). In order to increase the probability of the development of psychiatric syndromes, a stratified subsample of 591 subjects (292 males; 299 females) was selected for interview, with two-thirds consisting of high-scorers, that is, people with increased psychopathological symptom load as defined by the 85th percentile or more of the Global Severity Index (GSI) of the SCL-90-R and a random sample of those with scores below the 85th percentile of the GSI. Such a two-phase approach consisting of initial screening and subsequent interview with a stratified subsample is an established procedure in epidemiological research (23). For more information, see also reference (24). The seven interview waves were conducted in 1979, 1981, 1986, 1988, 1993, 1999, and 2008. The participant flow is shown in Figure 1.

The Zurich Cohort Study was approved by the ethics committee of the canton of Zurich (KEK) as fulfilling all ethical, legal and data privacy protection requirements and is in strict accordance with the declaration of Helsinki of the World Medical Association. All participants gave their written informed consent. Due to strict data protection requirements based on Swiss legislation effective at the time of study inception, we cannot make the data freely available. However, full analysis scripts and original data outputs can be obtained from the first author upon request for validation and reproduction of the results.

### Instruments and measures

Interviews were conducted with the “Structured Psychopathological Interview and Rating of the Social Consequences of Psychological Disturbances for Epidemiology” (SPIKE) (24). This semi-structured interview was specifically developed for epidemiological surveys in psychiatry and captures socio-demography, environmental conditions, somatic syndromes, psychopathology and social functioning. The interview includes 29 sections corresponding to major organ systems, functions, and disorders: stomach, intestines, respiration, heart, circulation, back, headache, allergies, pain, sleep, appetite, menstruation, sexuality, panic, anxiety, phobias, hypochondriasis, depression, hypomania, obsessive-compulsive syndromes, posttraumatic stress, suicidality, and substance use disorders. Its excellent reliability and validity have been reported in detail previously (19, 25). The inter-rater reliability of the SPIKE showed κs of 0.89 and 0.91 for the symptoms of depression (including insomnia) and anxiety and of 0.90 for the corresponding syndromal diagnoses. Validity has been assessed by comparing physician ratings and medical records with the SPIKE diagnoses among 140 patients drawn from psychiatric clinics or social-psychiatric services in the canton of Zurich and from a local hospital.

All interviews were administered by trained and supervised clinical psychologists or psychiatrists. As described in detail elsewhere (26), the 12-month prevalence rates of mental disorders were assessed according to DSM criteria: 1979–1986 by DSM-III, 1988–1993 by DSM-III-R, and 1999–2008 by DSM-IV. This was the case for major depressive disorders, generalized anxiety disorder (GAD), panic disorder, all phobias, and substance-use disorder. Obsessive-compulsive disorder (OCD) was always diagnosed according to DSM-III-R. For the present study we collapsed episodes of unipolar and bipolar depression into a diagnosis of mood disorder. Panic disorder, specific phobia, social phobia and agoraphobia as well as OCD were combined into an umbrella diagnosis of anxiety disorder. Alcohol as well as drug abuse or dependence was collapsed into a single diagnosis of substance-use disorder.

The 12-month prevalence of sleep problems was graded according to frequency (number of symptomatic days during the past 12 months, thus 1–365) and associated distress (rated on a visual analog scale from 0 to 100) at each assessment wave. Sleep problems were present if at least one of the following 10 items was endorsed: (1) trouble to get up in the morning, (2) difficulties to fall asleep, (3) awakening during night, (4) early awakening, (5) worrying not being able to sleep at night (not assessed in 1979 and 1981), (6) partially too much sleep (not assessed in 1979 and 1981), (7) being tired very early in the evening (not assessed in 1979 and 1981), (8) anxiety states during the night, (9) awakening with fear from nightmares (not assessed in 1979 and 1981), and (10) falling asleep unintentionally, e.g., during movies or meetings (not assessed in 1979 and 1981). We applied the following algorithm to provide a severity gradient: 12-month prevalence of sleep problems were defined as mild if frequency of any symptom was 1–7 days and associated distress 1–60, or if frequency 8–30 days and distress 1–30. A moderate syndrome was defined as frequency 1–7 days and distress 61–100, frequency 8–30 days and distress 31–60, or if frequency 31–365 and distress 1–30. Finally, a severe syndrome was defined as present if frequency 8–30 days and distress 61–100, if frequency 31–365 days and distress 31–100. Note that frequency and distress were not assessed for each symptom separately, but only for the presence of one of more symptoms. Disturbed sleep initiation was defined as present when the item “I had difficulties to fall asleep” was endorsed. Disturbed sleep initiation was chosen because it is a valid proxy of insomnia and the most researched specific sleep problem.

Twelve-month prevalence of suicidality was evaluated independently of depression, also at each wave based on five dichotomous items (i.e., present vs. absent): (1) I wouldn't mind being dead; (2) I had transient thoughts of harming myself; (3) I had serious persisting thoughts of harming myself; (4) I had clear and precise ideas of how to commit suicide; and (5) I made a suicide attempt. Items (1 and 2) were categorized as mild suicidality, and items (3–5) as severe suicidality. We preferred to assess both sleep problems and suicidality continuously (i.e., graded by severity), because it is now well established that psychopathology is dimensional by nature (27, 28). Assessing these syndromes as dichotomous diagnoses would result in loss of information and hence in a significant reduction of validity and reliability (29).

### Statistical analysis

For the longitudinal analyses of repeated measures of sleep problems and suicidality we fitted a series of Generalized Estimating Equations (GEE) (30), where sleep problems were included as the independent variable and suicidality as the dependent variable, though, worthy of note, these associations were concurrent and bi-directional. GEE models were introduced to fit regression analyses that account for within-subject correlation, which is an inherent part of longitudinal studies that rely on repeated outcome measures. The GEE approach uses weighted combinations between a predictor variable and repeated outcomes that account for varying observations, e.g., a disorder being present vs. absent, within a person across time. GEE use all available data and estimate missing values under the assumption of Missing Completely at Random (MCAR) (31). Prerequisite to the application of GEE was therefore a thorough missing value analysis, which revealed that both sleep problems and suicidality met the criteria of MCAR according to Little's MCAR test (χ^2^ = 322.4, *df* = 292, *p* = 0.107). Based on the probability density function of the outcome we applied multinomial distribution with cumulative logit link-function. The within-subject covariance was specified with the “unstructured” correlation type to avoid having any constraints on the covariance structure and a robust sandwich estimator was used to reduce the effects of outliers and influential observations. Prospective time-lagged associations were computed by relating the predictor variable at time point t to the outcome variable at t + 1. That is, sleep problems in 1979 were related to suicidality in 1981, sleep problems in 1981 to suicidality in 1986, and so on. In addition to examining the prospective effect of sleep problems on subsequent suicidality, we also tested for the reverse association, i.e., the prospective effect of suicidality at time point t on sleep problems at t + 1. All analyse were conducted with SPSS version 24 for Windows.

## Results

Across the whole 30-year observation period the final retention rate was 57%. The initial distribution above and below the 85th percentile of the GSI did not change over the seven interview waves, although dropouts were more common among extremely high or low scorers on the GSI (32). We repeated those dropout analyses for the last interview in 2008 and found that dropouts did not differ significantly in their socio-economic status and education at onset of the study from subjects who remained in the study. Neither was there a difference in initial psychopathologic impairment according to the nine SCL-90-R subscales, but there were slightly more dropouts among males (*OR* = 1.82; 95%-CI = 1.31–2.53; *p* < 0.001).

**Figure 2:**
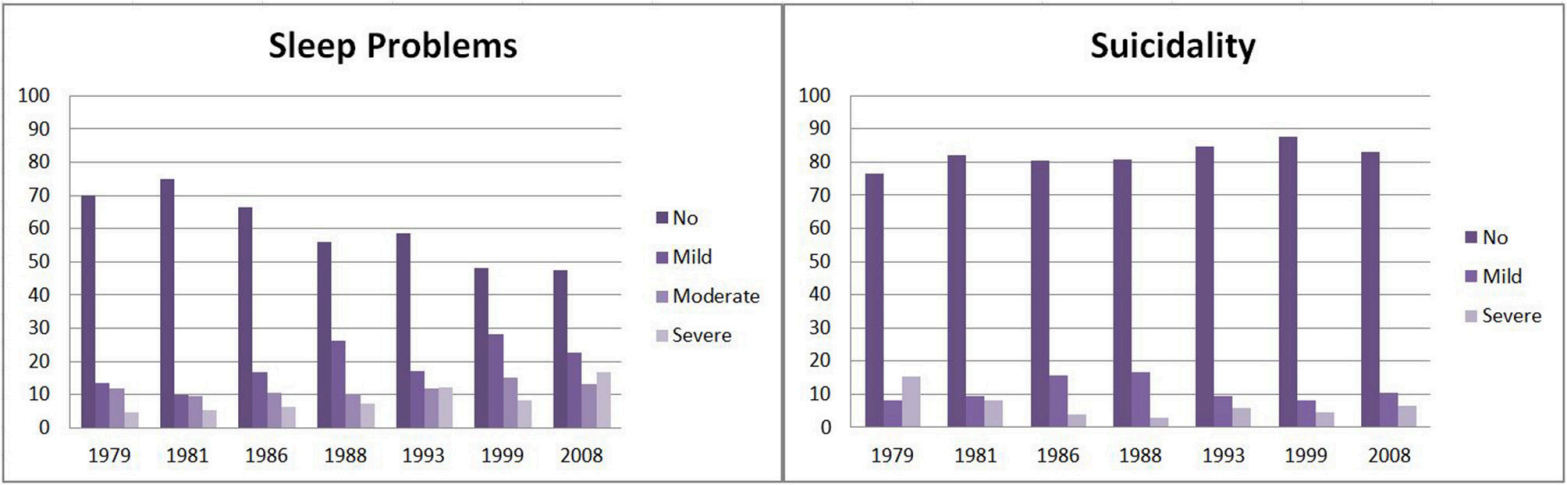
Prevalence of suicidality and sleep problems across a 30-year observation period.

A full description of all variables included in this paper is given in the Supplementary Table 1. The development of both sleep problems and suicidality from age 20/21 to 49/50 is indicated in Figure 2. The prevalence of both sleep problems and suicidality varied significantly across time (both *p* < 0.001). The prevalence of sleep problems increased steadily with age, while suicidality decreased from age 20/21 to 40/41 and increased afterwards again to the age 49/50. According to bivariate GEE analyses, both sleep problems (*OR* = 1.46, 95%-*CI* = 1.21–1.76, *p* < 0.001) and suicidality (*OR* = 1.34, 95%-*CI* = 1.03–1.75, *p* = 0.014) were slightly more prevalent in women. Having children increased the risk of sleep problems (*OR* = 1.41, 95%-*CI* = 1.20–1.66, *p* < 0.001), but reduced the risk of being suicidal (*OR* = 0.58, 95%-*CI* = 0.46–0.74, *p* < 0.001). Sleep problems were significantly related to concurrent mood disorders (*OR* = 2.79, 95%-*CI* = 2.27–3.43), anxiety disorders (*OR* = 2.29, 95%-*CI* = 1.92–2.72), and substance-use disorders (*OR* = 1.54, 95%-*CI* = 1.23–1.92). Likewise, suicidality was strongly related to concurrent mood disorder (*OR* = 4.71, 95%-*CI* = 3.59–6.19), and, to a lesser extent, to anxiety (*OR* = 2.15, 95%-*CI* = 1.69–2.73) and substance-use (*OR* = 1.55, 95%-*CI* = 1.18–2.04) disorders. These preliminary results emphasize the need to adjust for these important confounders.

We then tested the concurrent bi-directional associations between sleep problems and suicidality longitudinally across time (Table 1). It is shown that sleep problems relate to increased suicidality, with mild sleep problems relating to more suicidality than no sleep problems, and moderate sleep problems relating to higher suicidality than mild sleep problems. However, severe sleep problems did not significantly differ from moderate sleep problems. This pattern held true in the simple bivariate model (model 1) as well as when adjusted for socio-demographics (model 2) and concomitant mood, anxiety and substance-use disorders (model 3). Interaction effects between sleep problems and either mood, anxiety, or substance-use disorders or sex were not significant (all *p* > 0.18).

**Table 1:** Longitudinal concurrent associations between sleep problems and suicidality.

**Models and included predictor variables**		**Model effects *X*^2^(*df*); *p***	**Estimates of sleep problems**	***OR***	**95% *CI***
1:	Sleep problems	97.4(3); *p* < 0.001	Severe Moderate Mild No	2.46 3.61 1.71 Ref.	1.74; 3.46 2.75; 4.73 1.31; 2.24
2:	Sleep problems Time/age Sex Education Parental income Children	92.3 (3); *p* < 0.001 28.2 (6); *p* < 0.001 6.5 (1); *p* = 0.011 0.2 (2); *p* = 0.890 1.6 (2); *p* = 0.449 4.5 (1); *p* = 0.034	Severe Moderate Mild No	2.85 3.60 1.95 Ref.	1.98; 4.10 2.70; 4.82 1.49; 2.54
3:	Sleep problems Time/age Sex Education Parental income Children Mood disorder Anxiety disorder SUD	72.6 (3); *p* < 0.001 50.9 (6); *p* < 0.001 2.0 (1); *p* = 0.159 0.6 (2); *p* = 0.742 2.3 (2); *p* = 0.321 2.9 (1); *p* = 0.091 109.5 (1); *p* < 0.001 21.1 (1); *p* < 0.001 10.3 (1); *p* = 0.001	Severe Moderate Mild No	1.87 3.27 1.88 Ref.	1.26; 2.79 2.47; 4.35 1.42; 2.49

Next we tested prospective associations, that is, whether one syndrome at time t was able to predict the other syndrome at t + 1. We first examined, whether sleep problems prospectively predicted subsequent suicidality, but found no effect in the unadjusted model (Wald χ^2^ = 3.8, *df* = 3, *p* = 0.287) and all others (all *p* > 0.2). In a second step we examined whether suicidality would prospectively predict subsequent sleep problems, which consistently yielded significant results (Table 2). However, irrespective of the control variables included in the model (see model 1–3), only mild suicidality prospectively predicted sleep problems, but not severe suicidality.

**Table 2:** Longitudinal prospective effects of suicidality (predictor) on subsequent sleep problems (outcome).

**Models and included predictor variables**		**Model effects *X*^2^ (*df*); *p***	**Estimates of suicidality**	***OR***	**95% *CI***
1:	Suicidality	6.8 (2); *p* = 0.033	Severe Mild No	0.91 1.35 Ref.	0.65; 1.28 1.06; 1.72
2:	Suicidality Time/age Sex Education Parental income Children	11.3 (2); *p* = 0.003 66.4 (5); *p* < 0.001 8.4 (1); *p* = 0.004 4.8 (2); *p* = 0.089 1.0 (2); *p* = 0.594 0.1 (1); *p* = 0.792	Severe Mild No	1.16 1.53 Ref.	0.81; 1.66 1.19; 1.97
3:	Suicidality Time/age Sex Education Parental income Children Mood disorder Anxiety disorder SUD	8.9 (2); *p* = 0.012 56.8 (5); *p* < 0.001 9.4 (1); *p* = 0.002 5.0 (2); *p* = 0.084 1.0 (2); *p* = 0.600 (1); *p* = 0.742 2.2 (1); *p* = 0.137 0.3 (1); *p* = 0.577 4.4 (1); *p* = 0.037	Severe Mild No	1.04 1.47 Ref.	0.72; 1.51 1.14; 1.90

We additionally ran sensitivity analyses where we used disturbed sleep initiation (coded as present vs. absent) instead of broad sleep problems (coded according to severity). The bi-directional associations were substantial and almost identical to those reported in Table 1 (data upon request). The prospective effect of disturbed sleep initiation on subsequent suicidality was significant in both the unadjusted crude model and when adjusted for socio-demographics and concomitant mental disorders (Table 3). Moreover, consistent with the findings concerning broad sleep problems detailed above, mild suicidality, but not severe suicidality, prospectively related to subsequent disturbed sleep initiation (Table 4).

**Table 3:** Longitudinal prospective effects of disturbed sleep initiation (predictor) on subsequent suicidality (outcome).

**Models and included predictor variables**		**Model effects *X*^2^ (*df*); *p***	**Estimates of DSI**	***OR***	**95% *CI***
1:	Disturbed sleep initiation	10.3 (1); *p* = 0.001	Yes No	1.50 Ref.	1.17; 1.92
2:	Disturbed sleep initiation Time/age Sex Education Parental income Children	8.5 (1); *p* = 0.004 16.5 (5); *p* = 0.006 8.2 (1); *p* = 0.004 3.1 (2); *p* = 0.217 2.0 (2); *p* = 0.363 3.1 (1); *p* = 0.078	Yes No	1.51 Ref.	1.14; 1.99
3:	Disturbed sleep initiation Time/age Sex Education Parental income Children Mood disorder Anxiety disorder SUD	7.5 (1); *p* = 0.006 19.9 (5); *p* = 0.001 7.9 (1); *p* = 0.005 3.5 (2); *p* = 0.173 2.3 (2); *p* = 0.314 3.2 (1); *p* = 0.072 0.9 (1); *p* = 0.333 2.1 (1); *p* = 0.151 1.6 (1); *p* = 0.203	Yes No	1.47 Ref.	1.12; 1.93

*DSI, Disturbed Sleep Initiation*.

**Table 4:** Longitudinal prospective effects of suicidality (predictor) on subsequent disturbed sleep initiation (outcome).

**Models and included predictor variables**		**Model effects *X*^2^ (*df*); *p***	**Estimate of suicidality**	***OR***	**95% *CI***
1:	Suicidality	14.2 (2); *p* = 0.001	Severe Mild No	1.08 1.61 Ref.	0.74; 1.58 1.26; 2.07
2:	Suicidality Time/age Sex Education Parental income Children	11.5 (2); *p* = 0.003 47.8 (5); *p* < 0.001 1.1 (1); *p* = 0.287 3.5 (2); *p* = 0.171 1.0 (2); *p* = 0.595 1.1 (1); *p* = 0.297	Severe Mild No	1.21 1.56 Ref.	0.81; 1.81 1.20; 2.03
3:	Suicidality Time/age Sex Education Parental income Children Mood disorder Anxiety disorder SUD	8.3 (2); *p* = 0.016 46.3 (5); *p* < 0.001 0.9 (1); *p* = 0.338 3.6 (2); *p* = 0.163 1.1 (2); *p* = 0.578 1.2 (1); *p* = 0.264 1.8 (1); *p* = 0.175 0.5 (1); *p* = 0.480 0.5 (1); *p* = 0.495	Severe Mild No	1.11 1.49 Ref.	0.73; 1.68 1.14; 1.96

## Discussion

The research question under investigation here focuses on the relationship between sleep problems and suicidality. The data basis for this analysis was a longitudinal cohort study with seven assessment waves over a 30-year time period in a representative community sample of adults at increased risk of mental disorders. The longitudinal long-term design of our study offers a unique opportunity to analyse the complex relationship between sleep problems and suicidality exceeding previous analyses which rely mostly on cross-sectional data or short-term longitudinal studies. In contrast to previous studies on this subject, which relied on narrowly defined clinical samples [e.g., (8, 9, 14)], the present longitudinal study is based on a less restrictive population-based community sample.

At first sight, looking at the relationship between sleep problems and suicidality with respect to age, there seems to be a negative correlation: on a population level the prevalence rates of sleep problems increase steadily with age while the prevalence rates of suicidality decrease from age 20/21 to 40/41 with a small increase at age 49/50. This is not so on an individual level. Based on a series of GEE, we found that the more severe the sleep problems are, the more pronounced is suicidality, but severe sleep problems did not relate to increased suicidality relative to moderate sleep problems both unadjusted and when adjusted for socio-demographic and mental disorders. We cannot make conclusions about the direction of this relationship, but a clinician would know that both directions are plausible as sleepless people tend to ruminate, and doubtless suicidal ideas will lead to sleep problems (10). Most importantly, the association between sleep problems and suicidality persists even if we control for socio-demographics and in particular for accompanying mood, anxiety, or substance-use disorders. This clarifies that sleep problems are not just an accompanying symptom of underlying mental disorders, but stand for themselves (12).

Analyzing the longitudinal relationship over a 30-year time period we could not identify a significant prospective effect of broad sleep problems on subsequent suicidality as reported by others based on clinical samples (9, 14), but surprisingly identified a reversed relationship, i.e., preceding suicidality on subsequent sleep problems. This is the more surprising as the time intervals analyzed reach from 2 to 10 years. However, it needs to be considered that only mild suicidality prospectively related to subsequent broad sleep problems and disturbed sleep initiation, but not severe suicidality. This is an unexpected finding that warrants further examination. A possible explanation is that mild suicidality, i.e., suicidal ideation, is a rather enduring, trait-like phenomenon, whereas severe suicidality, i.e., self-harm and suicide attempts, is a transient, time-limiting phenomenon.

In contrast to broad sleep problems, when we break down the syndrome to a single symptom of insomnia, we found a relationship between disturbed sleep initiation and subsequent suicidality as reported in the literature (9, 14, 15). This effect is significant when controlled in particular for concomitant mental disorders, which bolsters the view expressed above that sleep problems have public health significance independent of mental disorders and that they should be assessed in addition to psychopathology. These findings are consistent with a previous study using the same data which found a prospective effect of insomnia on subsequent depression (19). Thus, it appears that disturbed sleep initiation, or insomnia, does not only precede depression symptoms generally, but also specifically suicidality, which is a marker of severe depression.

We acknowledge the following limitations: Firstly, though carefully evaluated with semi-structured interviews, all measures applied in this study basically relied on self-report. Laboratory-based data on sleep characteristics are certainly worthwhile to include in future epidemiologic research. Secondly, we were not able to examine possible causal mechanisms. To establish etiological pathways, future research needs to carefully assess neurobiological and psychosocial mediators. Thirdly, the time gap between certain assessment waves was up to 10 years in the present study, which conveys some uncertainty due to multiple uncontrolled confounders that could have occurred during those long time periods.

## Conclusions

From a clinical point of view our study makes clear how important it is to have a close eye on both the syndromes of sleep disturbances and suicidality. Though the two syndromes are inter-related, there is not a clear linear-by-linear association, since, for unknown reasons, sleep problems in general and disturbed sleep initiation specifically, appears to relate more consistently to mild suicidality than to severe suicidality. Differentiating symptoms such as suicidal ideation and rumination from acute self-harm and suicide attempts could therefore constitute an interesting avenue for future research. A clinician always should address both syndromes in therapy as obviously sleep problems, in particular symptoms of insomnia, can aggravate suicidality and suicidality can have a deleterious effect on sleep. Securing sleep could decrease suicidality and therapeutically addressing suicidality should improve subsequent sleep problems. All in all, we can say that there seems to be an underlying pathomechanism, which establishes such a relationship not only on the short term but partly also on the long run.

## Author contributions

The manuscript represents original work which has not been submitted or published elsewhere. WR, JA, VA-G, and MH made substantial contributions in the acquisition of the data. MH and WR analyzed the data. All authors have participated in the concept and drafting of the article, in the interpretation of the data and in revising the article critically in regards to its intellectual content. All authors approved the final version of the manuscript and take public responsibility for its content.

### Conflict of interest statement

The authors declare that the research was conducted in the absence of any commercial or financial relationships that could be construed as a potential conflict of interest.
