# Supplementary material for: Sleep Disturbances and Suicidality–A Longitudinal Analysis From a Representative Community Study Over 30 Years
Source: Front Psychiatry. 2018 Jul 16;9:320. doi: 10.3389/fpsyt.2018.00320 (PMC6054984; doi:10.3389/fpsyt.2018.00320)
Supplement: Supplementary file 1 [file Table_1.docx]

Supplementary Table 1

*Descriptive statistics of all variables included in the analysis*

| **Categorical variables** | **Outcome**  **category** | **N** | **%** |
| --- | --- | --- | --- |
| Sex | Men  Women | 292  299 | 49.4  50.6 |
| Parental income 1979 | Low  Medium  High | 203  131  233 | 35.8  23.1  41.1 |
| Education level 1979 | Low  Medium  High | 161  205  201 | 28.4  36.2  35.4 |
| Children 1979 | No  Yes | 576  15 | 97.5  2.5 |
| Children 1981 | No  Yes | 420  36 | 92.1  7.9 |
| Children 1986 | No  Yes | 334  123 | 73.1  26.9 |
| Children 1988 | No  Yes | 271  153 | 63.9  36.1 |
| Children 1993 | No  Yes | 183  224 | 45.0  55.0 |
| Children 1999 | No  Yes | 124  241 | 34.0  66.0 |
| Children 2008 | No  Yes | 78  223 | 25.9  74.1 |
| Sleep problems 1979 | No  Mild  Moderate  Severe | 414  80  70  27 | 70.1  13.5  11.8  4.6 |
| Sleep problems 1981 | No  Mild  Moderate  Severe | 342  45  44  25 | 75.0  9.9  9.6  5.5 |
| Sleep problems 1986 | No  Mild  Moderate  Severe | 303  77  48  29 | 66.3  16.8  10.5  6.3 |
| Sleep problems 1988 | No  Mild  Moderate  Severe | 238  111  44  31 | 56.1  26.2  10.4  7.3 |
| Sleep problems 1993 | No  Mild  Moderate  Severe | 238  70  49  50 | 58.5  17.2  12.0  12.3 |
| Sleep problems 1999 | No  Mild  Moderate  Severe | 177  104  56  30 | 48.2  28.3  15.3  8.2 |
| Sleep problems 2008 | No  Mild  Moderate  Severe | 159  76  44  56 | 47.5  22.7  13.1  16.7 |
| Disturbed sleep initiation 1979 | Absent  Present | 442  149 | 74.8  25.2 |
| Disturbed sleep initiation 1981 | Absent  Present | 365  91 | 80.0  20.0 |
| Disturbed sleep initiation 1986 | Absent  Present | 295  162 | 64.6  35.4 |
| Disturbed sleep initiation 1988 | Absent  Present | 256  168 | 60.4  39.6 |
| Disturbed sleep initiation 1993 | Absent  Present | 290  117 | 71.3  28.7 |
| Disturbed sleep initiation 1999 | Absent  Present | 243  124 | 66.2  33.8 |
| Disturbed sleep initiation 2008 | Absent  Present | 245  90 | 73.1  26.9 |
| Suicidality 1979 | No  Mild  Severe | 453  47  91 | 76.6  8.0  15.4 |
| Suicidality 1981 | No  Mild  Severe | 375  44  37 | 82.2  9.6  8.1 |
| Suicidality 1986 | No  Mild  Severe | 367  72  18 | 80.3  15.8  3.9 |
| Suicidality 1988 | No  Mild  Severe | 342  70  12 | 80.7  16.5  2.8 |
| Suicidality 1993 | No  Mild  Severe | 345  38  24 | 84.8  9.3  5.9 |
| Suicidality 1999 | No  Mild  Severe | 321  30  16 | 87.5  8.2  4.4 |
| Suicidality 2008 | No  Mild  Severe | 278  35  22 | 83.0  10.4  6.6 |
| Mood disorder 1979 | Absent  Present | 547  44 | 92.6  7.4 |
| Mood disorder 1981 | Absent  Present | 412  44 | 90.4  9.6 |
| Mood disorder 1986 | Absent  Present | 410  47 | 89.7  10.3 |
| Mood disorder 1988 | Absent  Present | 376  48 | 88.7  11.3 |
| Mood disorder 1993 | Absent  Present | 352  55 | 86.5  13.5 |
| Mood disorder 1999 | Absent  Present | 317  50 | 86.4  13.6 |
| Mood disorder 2008 | Absent  Present | 294  41 | 87.8  12.2 |
| Anxiety disorder 1979 | Absent  Present | 526  65 | 89.0  11.0 |
| Anxiety disorder 1981 | Absent  Present | 419  37 | 91.9  8.1 |
| Anxiety disorder 1986 | Absent  Present | 381  76 | 83.4  16.6 |
| Anxiety disorder 1988 | Absent  Present | 321  103 | 75.7  24.3 |
| Anxiety disorder 1993 | Absent  Present | 301  106 | 74.0  26.0 |
| Anxiety disorder 1999 | Absent  Present | 281  86 | 76.6  23.4 |
| Anxiety disorder 2008 | Absent  Present | 269  66 | 80.3  19.7 |
| Substance use disorder 1979 | Absent  Present | 559  32 | 94.6  5.4 |
| Substance use disorder 1981 | Absent  Present | 426  30 | 93.4  6.6 |
| Substance use disorder 1986 | Absent  Present | 373  84 | 81.6  18.4 |
| Substance use disorder 1988 | Absent  Present | 365  59 | 86.1  13.9 |
| Substance use disorder 1993 | Absent  Present | 350  57 | 86.0  14.0 |
| Substance use disorder 1999 | Absent  Present | 306  61 | 83.4  16.6 |
| Substance use disorder 2008 | Absent  Present | 266  69 | 79.4  20.6 |
